# Central administration of afzelin extracted from *Ribes fasciculatum* improves cognitive and memory function in a mouse model of dementia

**DOI:** 10.1038/s41598-021-88463-6

**Published:** 2021-04-28

**Authors:** So-Young Oh, Min Jun Jang, Yun-Hyeok Choi, Hongik Hwang, Hyewhon Rhim, Bonggi Lee, Chun Whan Choi, Min Soo Kim

**Affiliations:** 1 Brain Science Institute, Korea Institute of Science and Technology (KIST), Seoul, Republic of Korea; 2 Natural Product Research Team, Gyeonggi Biocenter, Gyeonggido Business and Science Accelerator, Gyeonggi-Do, Republic of Korea; 3 Division of Bio-Medical Science & Technology, University of Science and Technology KIST School, Seoul, Republic of Korea; 4 Department of Food Science and Nutrition, Pukyong National University, Busan, Republic of Korea; 5 Infectious Disease Research Center, Citizen’s Health Bureau, Seoul Metropolitan Government, 110, Sejong-daero, Jung-gu, Seoul, 04524 Republic of Korea

**Keywords:** Biochemistry, Drug discovery, Neuroscience

## Abstract

Neurodegenerative disorders are characterized by the decline of cognitive function and the progressive loss of memory. The dysfunctions of the cognitive and memory system are closely related to the decreases in brain-derived neurotrophic factor (BDNF) and cAMP response element-binding protein (CREB) signalings. *Ribes fasciculatum,* a medicinal plant grown in diverse countries, has been reported to pharmacological effects for autoimmune diseases and aging recently. Here we found that afzelin is a major compound in *Ribes fasciculatum*. To further examine its neuroprotective effect, the afzelin (100 ng/µl, three times a week) was administered into the third ventricle of the hypothalamus of C57BL/6 mice for one month and scopolamine was injected (i.p.) to these mice to impair cognition and memory before each behavior experiment. The electrophysiology to measure long-term potentiation and behavior tests for cognitive and memory functions were performed followed by investigating related molecular signaling pathways. Chronic administration of afzelin into the brain ameliorated synaptic plasticity and cognitive/memory behaviors in mice given scopolamine. Studies of mice’s hippocampi revealed that the response of afzelin was accountable for the restoration of the cholinergic systems and molecular signal transduction via CREB-BDNF pathways. In conclusion, the central administration of afzelin leads to improved neurocognitive and neuroprotective effects on synaptic plasticity and behaviors partly through the increase in CREB-BDNF signaling.

## Introduction

Neurodegenerative disease, such as Alzheimer’s disease (AD) which accounts for the largest portion of dementia cases, is characterized by a decline in cognitive function and the progressive loss of memory resulting from impairment in hippocampal neurogenesis^1–4^. The main pathological hallmarks of AD are amyloid plaques produced from deposits of amyloid β-proteins (Aβ) and neurofibrillary tangles caused by the aggregation of hyperphosphorylated tau proteins^5–7^. Neuroinflammation, oxidative stress, impaired synaptic plasticity, and cholinergic dysfunction are also components of the AD pathology^6,8–10^. In clinical trials, cholinergic drugs have been used for AD therapy by increasing acetylcholine (ACh) levels in the hippocampus^11–13^. These drugs, however, have displayed various side effects and overall low efficacy^14,15^. AD has also been associated with the down-regulation of brain-derived neurotrophic factor (BDNF) and its signaling pathways, potential contributing factors of Aβ deposition^16,17^. For the safer treatment of AD, compounds derived from natural products that simultaneously target multiple pathological components may be a promising method for the treatment of AD.

*Ribes fasciculatum* var. chinense MAX. (Saxifragaceae) grows widely in Korea, Japan, China, and various other countries^18–20^. Recently, *R. fasciculatum* has been reported to pharmacological effects on autoimmune diseases and aging-related disorders^20,21^, although there is little research about its effects on neurodegenerative disorders. Our group also demonstrated that the extract from the *R. fasciculatum* exhibited neuroprotective effects in a hydrogen peroxide-induced SH-SY5Y cell line^22^. Previous studies focused on in vitro experiments such as measuring chemical activities or working in cell lines. Here, we tried to confirm neuroprotective effects of afzelin derived from *R. fasiculatum* on scopolamine-injected mice.

Scopolamine is a non-selective blocker of muscarinic acetylcholine receptors, which consequently impairs learning and short-term memory, promoting amnesia^23,24^. Therefore, scopolamine-induced amnesia models are widely used to test the neuroprotective effects of natural products and related compounds. In this study, we performed electrophysiology and behavior tests to investigate the neuroprotective effect of afzelin on the cognitive function of a scopolamine-injected mouse model.

## Results

### Structural identification of Afzelin from *R. fasciculatum*

**Figure 1:**
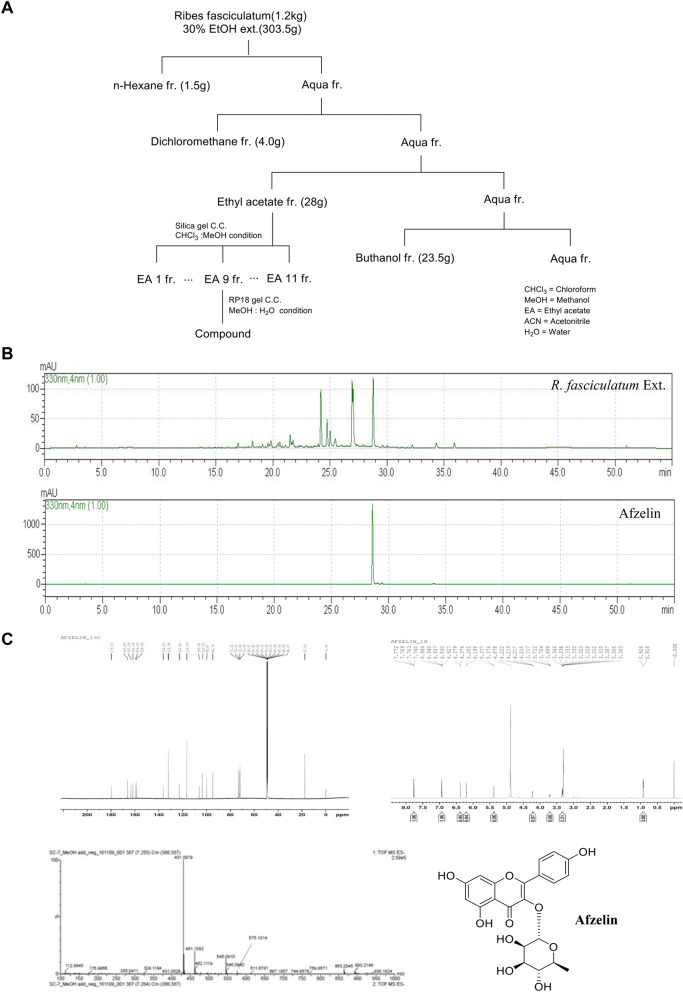
Isolation and structural analysis of afzelin from *Ribes fasciculatum* extract. Isolation scheme of compound from *R. fasciculatum* (**A**), HPLC profile of *R. fasciculatum* Extract and afzelin (**B**), ^13^C-NMR (CD_3_OD, 175 MHz, DMSO-d_6_), ^1^H-NMR (CD_3_OD, 700 MHz, DMSO-d_6_) and mass spectrum of afzelin from the *R. fasciculatum* (**C**).

Isolation procedures of afzelin from *R. fasciculatum* are summarized in Fig. 1A. *R. fasciculatum* (1.2 kg) was extracted with 30% EtOH. After completion of extraction, the solution was concentrated under reduced pressure to obtain 303.5 g of EtOH extract. The extract was separated with n-hexane (1.5 g), CH_2_Cl_2_ (4.0 g), EtOAc (28.0 g), and n-BuOH (23.5 g) from water. Purification of the fraction of EtOAc by column chromatography on silica gel with MeOH/CHCl_3_ as eluent gave 11 subfractions. Among the 11 subfractions, subfraction 9 was purified by a preparative HPLC on RP18 gel with MeOH/H_2_O as eluent to afford the compound (21 mg). The HPLC results provided specific information on the major compound, afzelin, in *Ribes fasciculatum* (Fig. 1B).

We found that the mass value of the protonated compound was 431, which is identical to the calculated mass for C_21_H_20_O_10_ [M-H]^-^. As shown in ^1^H-NMR spectrum, we could observe four aromatic peaks on flavonoid (δ 7.76, δ 6.93, δ 6.37, and δ 6.20) as well as distinctive rhamnoside signals (δ 5.37 as an acetal and δ 0.92 as a methyl group). Finally, the structure of the isolated compound was identified as afzelin by comparison with spectroscopic data (Fig. 1C) and reported data^25^.

### The effective dose of afzelin on cognition and memory

**Figure 2:**
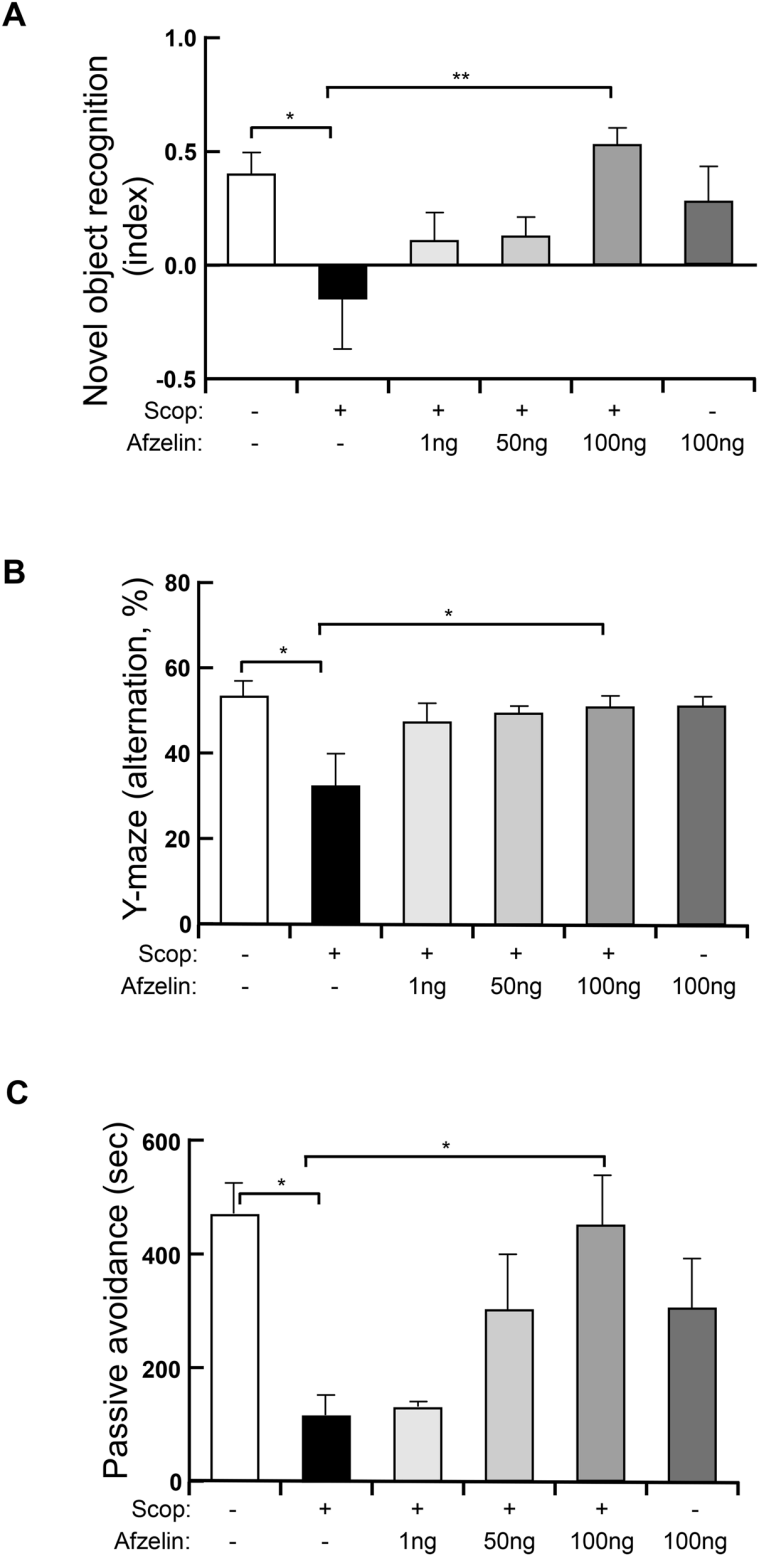
The optimal dose of afzelin in cognition and memory behaviors. Standard C57BL/6 mice (4 weeks old) were pre-treated into third-ventricle of brains cannula with specific doses (0.5 µl, 1, 50, 100 ng/µl) of Afzelin or PBS (0.5 µl) for one month. The mice were received with scopolamine (Scop) injection (i.p. 0.8 mg/kg) before each behavior test. (**A**–**C**) Novel object recognition tests (**A**), Y-maze tests (**B**) and passive avoidance tests (**C**) were conducted on the control (Scop −, Afzelin −), scop + vehicle (Scop +, Afzelin −), scop + 1 ng afzelin (Scop +, afzelin 1 ng), scop + 50 ng afzelin (Scop +, afzelin 50 ng), scop + 100 ng afzelin (Scop + , afzelin 100 ng) and afzelin alone (Scop –, Afzelin 100 ng) groups. *p < 0.05, **p < 0.01; one-way ANOVA with Tukey’s post hoc test (**A**–**C**); n = 4–6 mice for Novel object recognition (**A**), n = 5–7 mice for Y-maze (**B**), n = 4–5 mice for Passive avoidance (**C**). Data are mean ± s.e.m.

To figure out the optimal dose of afzelin, we applied the low to a high dose of afzelin (1, 50, 100 ng) into the third-ventricle of brains in experimental animals for one month. After pretreatment of afzelin over one month, we injected scopolamine into these animals before behavior tests (30 min before). In the tests of novel object recognition, the scopolamine significantly decreased novel recognitions of mice. The pretreatments of 1 and 50 ng afzelin with scopolamine injection did not increase cognition but the pretreatments of 100 ng afzelin with scopolamine injection only showed a significant increase of mice’s cognitions (Fig. 2A. F_(5, 23)_ = 3.552, Scop + vehicle vs. Scop + 100 ng afzelin p < 0.01). In addition, the administration of a single dose of afzelin (100 ng) without scopolamine injection showed no significant effect (Fig. 2A). To test spatial learning and memory, we conducted a Y-maze test for measuring spontaneous alteration. The pretreatments of afzelin (1 ng) with scopolamine injection did not increase the spatial learning and memory but the pretreatments of 50 and 100 ng afzelin with scopolamine injection showed a significant increase in the memory of mice (Fig. 2B. F_(5, 28)_ = 5.435, Scop + vehicle vs. Scop + 100 ng afzelin p < 0.05). The single administration of afzelin (100 ng) without scopolamine injection showed similar levels with control in the Y-maze test (Fig. 2B). In the passive avoidance test to understand the effect on memory by afzelin administration, the pretreatments of 1 and 50 ng of afzelin with scopolamine injection did not increase cognition but the pretreatments of 100 ng of afzelin with scopolamine injection only showed a significant increase in the memory of mice (Fig. 2C. F_(5, 21)_ = 5.156, Scop + vehicle vs. Scop + 100 ng afzelin p < 0.05). The administration of single afzelin (100 ng) without scopolamine injection showed that an increase in memory but the extent was not higher than the scop + 100 ng afzelin group (Fig. 2C). Based on these results, we decided on the effective dose of afzelin (100 ng) to improve mice’s cognition and memory. Therefore, we used central treatments of 100 ng afzelin for the next experiments.

### Afzelin rescues a deficit in HFS-induced LTP caused by scopolamine

**Figure 3:**
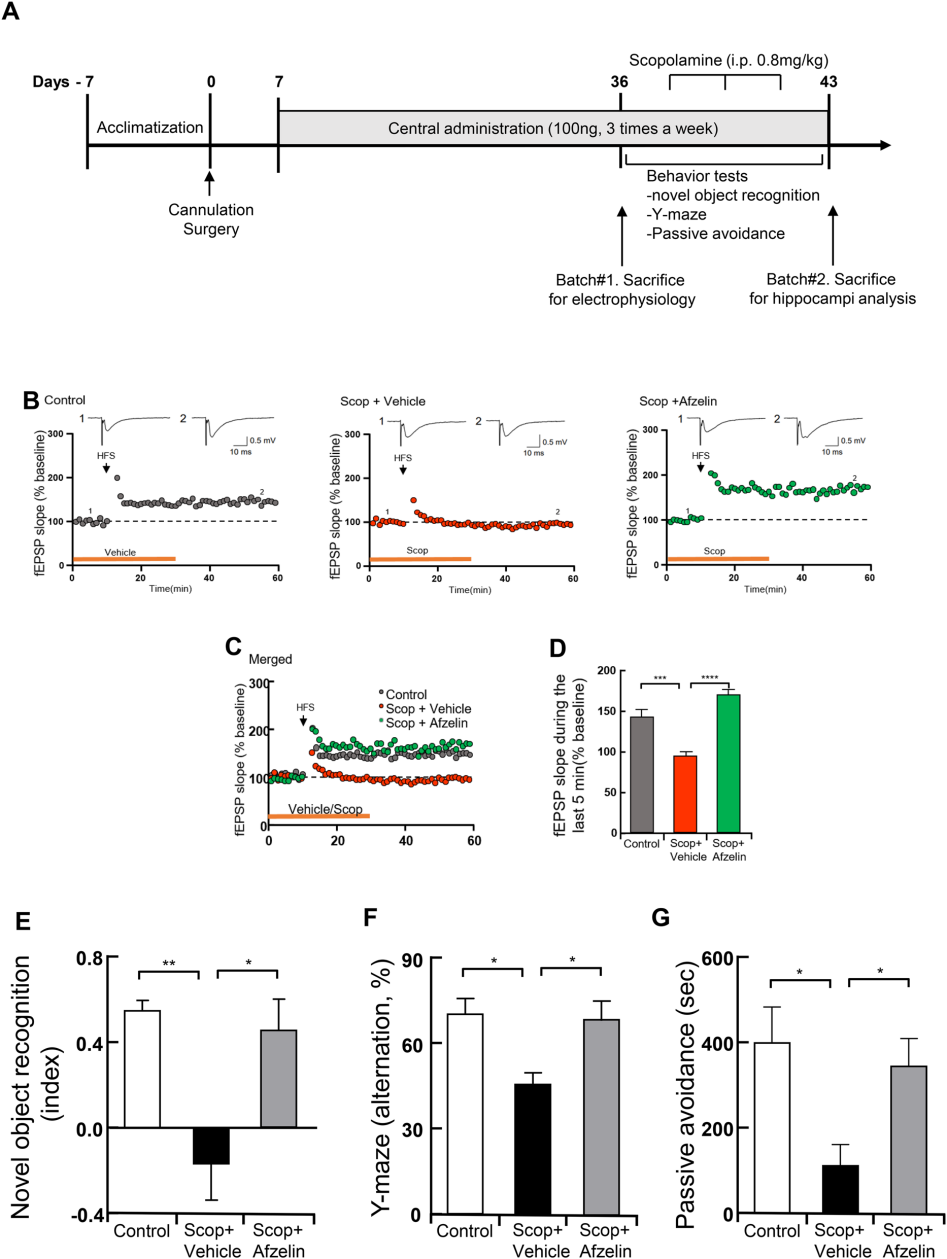
Chronic administration of afzelin exerts a protective effect against the Scop-induced deficit in hippocampal LTP, cognition, and memory. Standard C57BL/6 mice (4 weeks old) were administrated via pre-implanted third-ventricle cannula with a single dose (0.5 µl, 100 ng/µl) of Afzelin or PBS (0.5 µl) for one month. (**A**) the schematic timeline of the experiments. For the electrophysiology (batch#1), mice were sacrificed then their brain’ slices were treated with standard aCSF containing either DMSO or scopolamine (100 μM) to the control carrying DMSO (Control), scopolamine + DMSO (Scop + vehicle), or scopolamine + afzelin (Scop + Afzelin) groups during the baseline recording (10 min) and for additional 20 min after LTP induction by HFS. (**B**) A change in the fEPSP slope was monitored following LTP induction by high-frequency stimulation (HFS, arrow) at SC-CA1 synapses in the hippocampus. The magnitude of LTP was quantified as an increase in the fEPSP slope relative to the baseline. Filled circles indicate averaged fEPSPs at each time point, and the error bars represent ± SEM (scale bars, 0.5 mV, 10 ms). (**B**) Individual panels in (**C**) are merged for comparisons. (**D**) A bar graph representing the magnitude of LTP (% of baseline) during the last 5 min. For behavior tests of cognition and memory (batch#2), The mice were received with scopolamine (Scop) injection (i.p. 0.8 mg/kg) before 30 min for each behavior tests. (**E**–**G**) Novel object recognition tests (**E**), Y-maze tests (**F**) and passive avoidance tests (**G**) were conducted on the control (PBS as a vehicle), scopolamine + vehicle (Scop + vehicle, PBS as a vehicle) and scopolamine + Afzelin (Scop + Afzelin, 100 ng/µl afzelin of pretreatments) groups. *p < 0.05, **p < 0.01, ***p < 0.001, ****p < 0.0001; one-way ANOVA with Tukey’s post hoc test; n = 6 mice for the control group, n = 7 mice for Scop + vehicle, n = 6 mice for Scop + Afzelin (**B**–**D**), n = 5–7 mice for the behavior tests (**E**, **F**). *p < 0.05; one-way ANOVA with fisher's LSD test; n = 4–5 mice for Passive avoidance (**G**). Data are mean ± s.e.m.

Long-term potentiation (LTP), a long-lasting increase in the efficacy of excitatory synaptic transmission, is widely accepted as a cellular mechanism of learning and memory in the brain^26,27^. We examined whether the chronic administration of afzelin to the brain rescues the scopolamine-induced deficit in hippocampal LTP. For the measurement of hippocampal LTP, a single dose of afzelin (100 ng/µl, three times a week, for one month) was administered to C57BL/6 mice for a month via pre-implanted third-ventricle cannula. After pretreatments of afzelin for one month, we sacrificed mice and made the slices of the brain for LTP measurements. In all experiments, hippocampal slices were perfused with artificial cerebrospinal fluid (aCSF) containing either DMSO vehicle or scopolamine (100 μM) during the baseline recording and for an additional 20 min after LTP induction. In control mice, the high-frequency stimulation (HFS; 100 Hz for 1 s, three trains with 30-s interval) induced a robust increase in the excitatory postsynaptic potentials (fEPSP) slope (Fig. 3B, left panel; 143.6 ± 8.9%). However, the acute treatment with scopolamine severely impaired the induction of hippocampal LTP (Fig. 3B, middle panel; 95.4 ± 4.9%). Interestingly, the central injection of Afzelin fully recovered the scopolamine-induced impairment of hippocampal LTP (Fig. 3B, right panel; 170.4 ± 7.0%), indicating that the chronic administration of afzelin exerts a protective effect against the scopolamine-induced neurotoxicity in the hippocampus (Fig. 3C, D, D: F_(2, 16)_ = 31.37, Scop + vehicle vs. Scop + Afzelin, p < 0.0001).

### Central administration of afzelin recovered cognitive, learning, and memory behaviors

We further investigated whether the afzelin-mediated improvement of hippocampal LTP is associated with the cognitive, learning, and memory behavior changes in scopolamine-injected mice. For the behavior tests, C57BL/6 mice were administrated via pre-implanted third-ventricle cannula with a single dose (100 ng) of afzelin for a month. We conducted novel object recognition tests for evaluating cognition and recognition memory. Before performing the tests, we injected scopolamine into the vehicle- and afzelin-treated mice to impair cognition and memory as previously reported^8,28,29^. As expected, scopolamine injection markedly reduced cognition and memory functions but afzelin almost fully recovered them in the novel object recognition tests (Fig. 3E, F_(2, 13)_ = 8.413, Scop + vehicle vs. Scop + Afzelin, p < 0.05). To test spatial learning and memory, we conducted a Y-maze test for measuring spontaneous alteration. Compared to the vehicle-injected control group, central-administrated Afzelin led to the increase in learning and memory in the scopolamine-treated mice (Fig. 3F, F_(2, 16)_ = 6.294, Scop + vehicle vs. Scop + Afzelin, p < 0.05). Scopolamine injection destroyed memory function compared to the vehicle-treated group whereas Afzelin recovered it (Fig. 3G, F_(2, 11)_ = 5.349, Scop + vehicle vs. Scop + Afzelin, p < 0.05).

### Afzelin restored the dysfunction of the cholinergic system in the hippocampus

**Figure 4:**
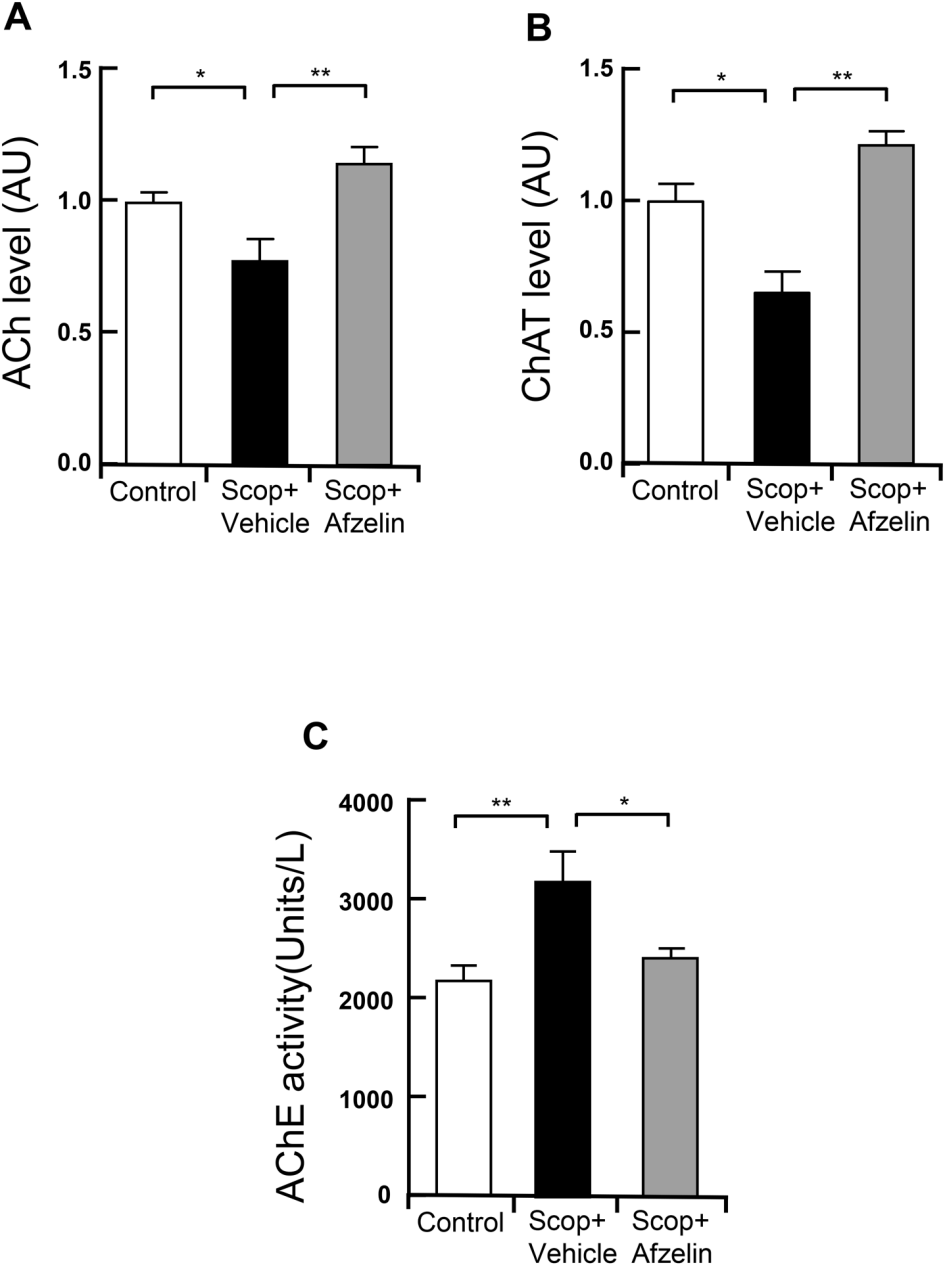
Effect of afzelin on cholinergic function in the hippocampus. Standard C57BL/6 mice (4 weeks old) were pre-treated into third-ventricle of brains cannula with 100 ng/µl of Afzelin (0.5 µl) or PBS (0.5 µl) for one month. The mice were received with scopolamine (Scop) injection (i.p. 0.8 mg/kg) for the behavior tests then hippocampi of these mice were collected for measuring cholinergic changes. (**A**–**C**) Acetylcholin (ACh) levels (**A**), Choline acetyltransferase (ChAT) level (**B**), Acetylcholinesterase (AChE) activity (**C**) in the hippocampus were measured in hippocampi of the control (PBS as a vehicle), scopolamine + vehicle (Scop + vehicle, PBS as a vehicle) and scopolamine + Afzelin (Scop + Afzelin, 100 ng/µl afzelin of pretreatments) groups *p < 0.05, **p < 0.01; one-way ANOVA with Tukey’s post hoc test (**A**–**C**); n = 3–4 mice for Acetylcholine (ACh) levels (**A**), n = 3 mice for Choline acetyltransferase (ChAT) level (**B**), n = 4–6 for Acetylcholinesterase (AChE) activity (**C**). Data are mean ± s.e.m.

We investigated whether the central administration of afzelin affects acetylcholine contents and the activities of choline acetyltransferase and acetylcholinesterase that represent the cholinergic systems associated with neural functions. The hippocampi of mice given scopolamine significantly decreased acetylcholine (ACh) contents and the levels of choline acetyltransferase (ChAT), and increased activity of acetylcholinesterase (AChE). In contrast, the chronically afzelin-treated hippocampi of mice given scopolamine significantly recovered contents of ACh and activities of ChAT, while activities of AChE were reduced (Fig. 4A–C, A: F_(2, 8)_ = 13.39, Scop + vehicle vs. Scop + Afzelin, p < 0.01, B: F_(2, 6)_ = 17.92, Scop + vehicle vs. Scop + Afzelin, p < 0.01, C: F_(2, 13)_ = 8.588, Scop + vehicle vs. Scop + Afzelin, p < 0.05), suggesting that afzelin protects scopolamine-induced deterioration of the cholinergic system.

### Afzelin induces neuroprotective effect via CREB-BDNF signaling

**Figure 5:**
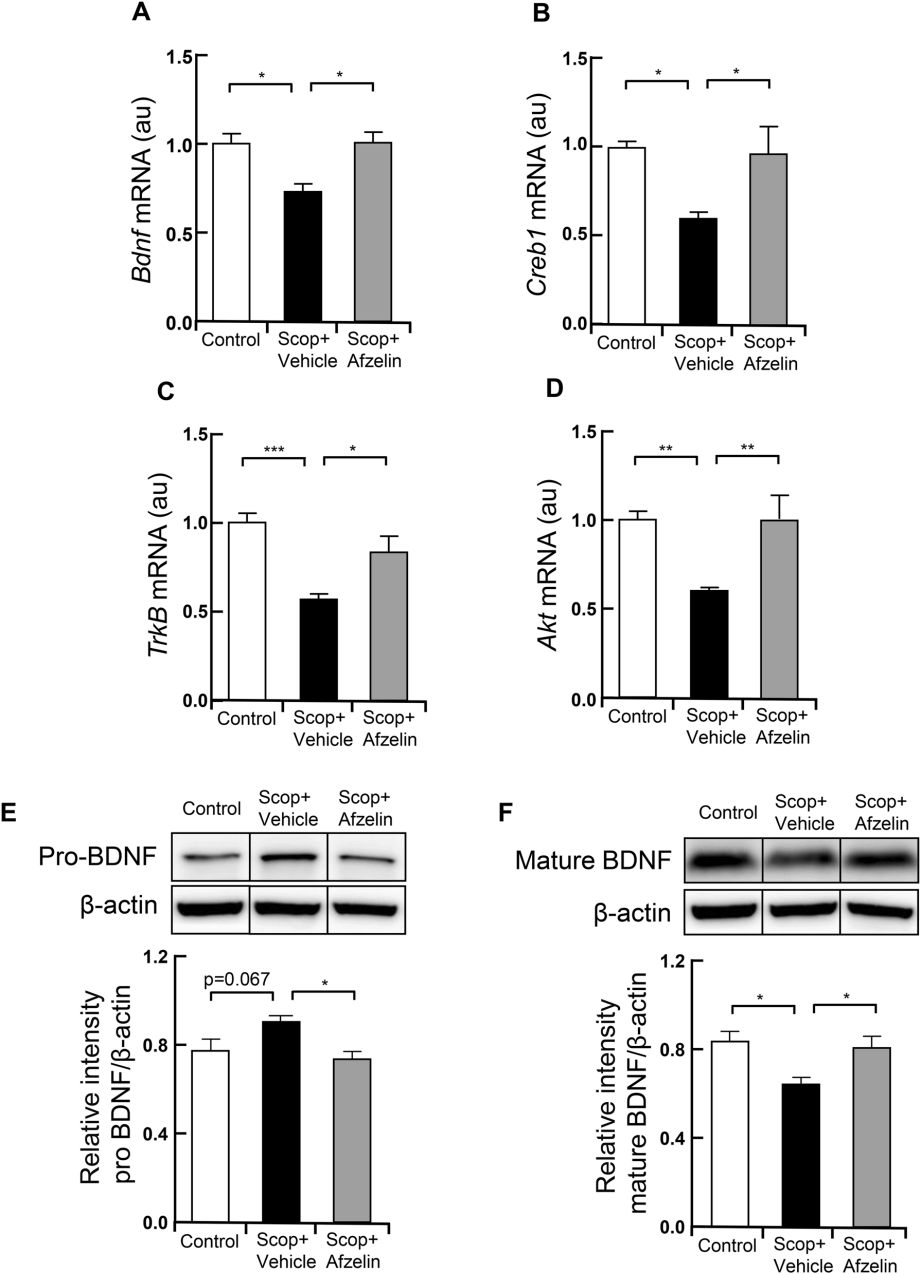
Alterations in the CREB-BDNF related signaling. Standard C57BL/6 mice (4 weeks old) were pre-treated into third-ventricle of brains cannula with 100 ng/µl of Afzelin (0.5 µl) or PBS (0.5 µl) for one month. The mice were received with scopolamine (Scop) injection (i.p. 0.8 mg/kg) for the behavior tests then hippocampi of these mice were collected for measuring mRNA and protein levels. (**A**–**D**) mRNA levels of BDNF (**A**), CREB (**B**), TrkB (**C**), and AKT (**D**) were analyzed using Real-Time RCR, and the protein levels of pro-BDNF (**E**) and mature BDNF (**F**) were measured using western blots with representative bands (black boxes from Suppl. Fig. S1) and their quantified bar graphs, which were cropped and analyzed from whole images in Supplementary Fig. S1, in the control (PBS as a vehicle), scopolamine + vehicle (Scop + vehicle, PBS as a vehicle) and scopolamine + Afzelin (Scop + Afzelin, 100 ng/µl afzelin of pretreatments) groups. *p < 0.05, **p < 0.01 ***p < 0.001; one-way ANOVA with Tukey’s post hoc test (**A**–**F**); n = 6 mice for qPCR (**A**–**D**) and n = 5 for western blotting (**E**, **F**). Data are mean ± s.e.m.

**Figure 6:**
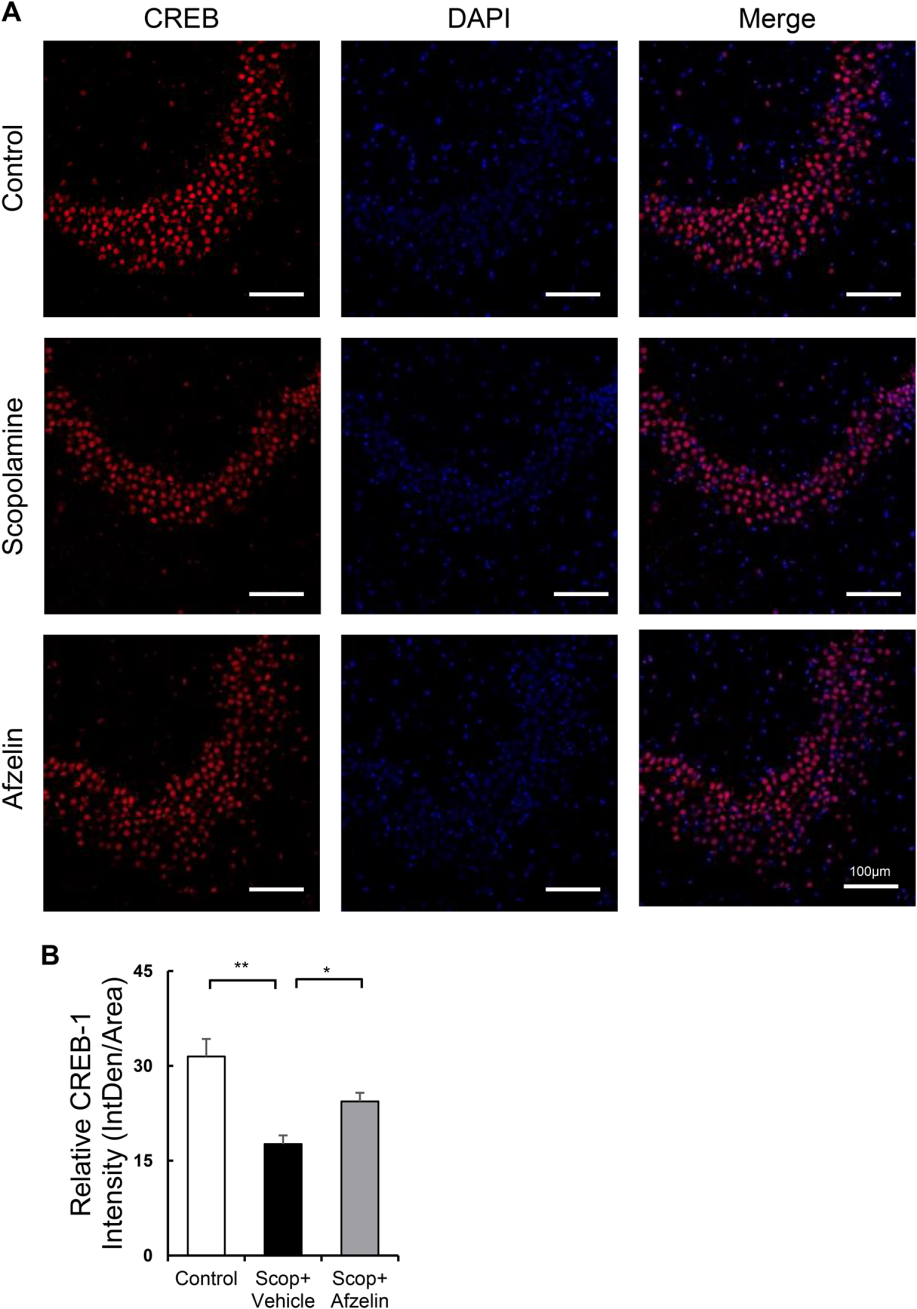
Immunofluorescence analysis of brain sections from the hippocampus. Standard C57BL/6 mice (4 weeks old) were pre-treated into third-ventricle of brains cannula with 100 ng/µl of Afzelin (0.5 µl) or PBS (0.5 µl) for one month. The mice were received with scopolamine (Scop) injection (i.p. 0.8 mg/kg) for the behavior tests then hippocampi of these mice were collected for immunofluorescence analysis. The representative images of immunoreactivity to CREB expression in the hippocampal CA3 region were shown with CREB (red) and DAPI (blue) using confocal microscopes (**A**). These images were quantified to bar graph in the control (PBS as a vehicle), scopolamine + vehicle (Scop + vehicle, PBS as a vehicle) and scopolamine + Afzelin (Scop + Afzelin, 100 ng/µl afzelin of pretreatments) groups (**B**). Scale bars, 100 μm. The fluorescence intensity was quantified using Image J. *p < 0.05 **p < 0.01; one-way ANOVA with Dunnett’s post hoc test; n = 3 for quantified bar graph (**B**). Data are mean ± s.e.m.

Previous studies have shown that scopolamine-induced impairment of memory and cognitive functions are closely associated with the down-regulation of the CREB-BDNF axis in the brain^8,30,31^. Thus, we determined whether the chronic afzelin treatment affects CREB /BDNF signaling. We isolated RNA from the hippocampus to analyze the mRNA expression of the BDNF signaling cascade. The hippocampus in the scopolamine injected group significantly decreased mRNA expression levels of BDNF but afzelin reversed it (Fig. 5A, F_(2, 15)_ = 6.963, Scop + vehicle vs. Scop + Afzelin, p < 0.05). Consistently, mRNA expression levels of BDNF downstream genes including TrkB, AKT, and CREB were decreased by scopolamine but reversed by afzelin treatments (Fig. 5B–D, B: F_(2, 15)_ = 5.322, Scop + vehicle vs. Scop + Afzelin, p < 0.05, C: F_(2, 15)_ = 11.89, Scop + vehicle vs. Scop + Afzelin, p < 0.05, D: F_(2, 15)_ = 8.238, Scop + vehicle vs. Scop + Afzelin, p < 0.01). The activity-dependent conversion of pro-BDNF to mature BDNF mediates synaptic competition^32^. Pro-BDNF selectively activates the p75 receptor to induce pro-apoptotic signaling, while mature BDNF binds with TrkB to promote neural regeneration and rehabilitation of cognition and memory^32,33^. The scop + vehicle groups showed increased levels of pro-BDNF proteins, compared to the control group. The scop + afzelin groups reduced the protein levels of pro-BDNF (Fig. 5E, F_(2, 12)_ = 5.665, Scop + vehicle vs. Scop + Afzelin, p < 0.05). The protein levels of mature BDNF decreased in the scop + vehicle groups but were recovered by afzelin treatments (Fig. 5F, F_(2, 12)_ = 6.241, Scop + vehicle vs. Scop + Afzelin, p < 0.05). To further investigate the effect of afzelin on BDNF-CREB signaling, we performed immunohistochemical analysis. The data showed significant expression of CREB (*Creb1*) in the hippocampus of mice given the central administration of afzelin compared to that of the scopolamine-injected group (Fig. 6A). When these images were quantified, greater numbers of CREB-positive-cells were observed in the hippocampus of mice given afzelin (Fig. 6B, F_(2, 6)_ = 12.93, Scop + vehicle vs. Scop + Afzelin, p < 0.05), indicating that the up-regulation of CREB-BDNF signaling may contribute to the afzelin-mediated amelioration of memory function impaired by scopolamine.

## Discussion

Afzelin is a flavonol glycoside natural product derived from kaempferol which has four hydroxy groups at positions 3, 5, 7, and 4′ on the flavone. The generation of a glycosidic bond between the 3-O atom of kaempferol and α-L-rhamnose produces the afzelin, thus there are 3 phenol groups and 5 stereogenic centers. Recently, afzelin has been evaluated to achieve various biological properties including antibacterial effect^34^ and high aldose reductase inhibitory activity^35^. In this report, we studied the neurocognitive and neuroprotective effects of afzelin isolated from *R. fasciculatum*.

We showed that the Afzelin, kaempferol 3-rhamnoside, improved cognitive and memory function in a scopolamine-injected mouse by performing the Y-maze and passive avoidance tests. These two behavior tests which are related to learning and memory function are involved with the region of hippocampal activities^36,37^. The underlying basis could be related to three functions of afzelin effects: induction of cholinergic changes, long-term potentiation (LTP), and BDNF/CREB expression signaling.

Scopolamine, a muscarinic acetylcholine receptor antagonist, is a commonly used chemical that impairs learning and memory in animal models to find therapeutic candidates for neurodegenerative diseases^38^. Because the effects of scopolamine treatment can induce cognitive deficit by decreasing acetylcholine contents and choline acetyltransferase, but increasing acetylcholinesterase activities. The afzelin might be considered a strong candidate for treating AD to recover acetylcholine cascades in the hippocampus with reduced symptoms^7,8,31^.

Long-term potentiation (LTP) represents a long-lasting increase in the efficacy of excitatory synaptic transmission, and it is widely used to measure a cellular mechanism of learning and memory in the brain^26,27^. Central administration of afzelin into the brain rescued the LTP in the mice which had destroyed memory function by scopolamine injection. The chronic administration to the brain with afzelin rescues the scopolamine-induced deficit in hippocampal LTP. The recovered capability of an action potential in the brain is important for brain protection in neurodegenerative diseases^39,40^.

BDNF in the hippocampal region is an essential growth factor for cell protection, synaptic transmission, and learning/memory functions^41–43^. The treatments with an anti-BDNF antibody or BDNF antisense mRNA induce memory impairments related to a reduction of LTP and ERK signaling^44–46^. The specific deletion of BDNF on the hippocampus impairs fear extinction, while hippocampal BDNF supports the acquisition of extinction memory^47^. Thus, BDNF is one of crucial factors in establishing fear extinction memory^46–48^. In addition, BDNF expression is controlled by CREB signaling in the hippocampus^49^. Activation of CREB facilitates the plasticity of BDNF as an activity-dependent protein^50^. The transcriptional induction of BDNF is dependent on phosphorylation of CREB, Ca2 + activated calmodulin kinase, or PKA, associated with improved spatial memory and induction of LTP^51–53^. Our data showed that the afzelin restored the down-regulation of BDNF and CREB expression in scopolamine-injected mice. Considering the importance of the BDNF-CREB axis in cognitive functions, we assume that the increase in BDNF-CREB signaling contributes to the afzelin-induced amelioration of learning and memory functions in scopolamine-injected mice. However, further studies are necessary to identify precise mechanisms underlying afzelin-mediated regulation of BDNF/CREB signaling related to cognitive function.

In conclusion, the central administration of afzelin ameliorates scopolamine-induced cognitive impairment in the brain. These effects of afzelin might be related to restored cholinergic dysfunction, enhanced LTP, and an increase of BDNF/CREB signaling.

## Methods

### Isolation of compounds from *Ribes fasciculatum* extract

The dried leaves of *Ribes fasciculatum* (1.2 kg) were extracted twice with 30% EtOH two times at room temperature and then filtered. The filtrate was evaporated under vacuum at 40 °C to obtain an EtOH extract (303 g), which was suspended in distilled water (4L) followed by partitioning with n-hexane (1.5 g), CH_2_Cl_2_ (4.0 g), EtOAc (28.0 g), and n-BuOH (23.5 g). The EtOAc soluble fraction(28.0 g) was separated by silica gel column chromatography using gradient mixtures as eluents [(Chloroform:MeOH; 100:0, 95:5, 90:10, 80:20, 70:30, 50:50, 30:70, 0:100)], (F001-011). F009 was purified by preparative HPLC (column: YMC-Pack ODS-A, 5 μm, 250 × 20 mm I.D., Japan, 8 mL/min, MeCN-H_2_O, 5:95 to 80:20, 60 min) to yield compound (Fig. 1A).

Yellow powder. ESI–MS m/z 431 [M−H]^−^. ^1^H-NMR (700 MHz, DMSO-d_6_) δ 7.75 (2H, d, *J* = 8.9 Hz, H-3′, 5′), 6.91 (2H, d, *J* = 8.8 Hz, H-2′, 6′), 6.36 (1H, dd, *J* = 2.0 & 14.4 Hz, H-8), 6.18 (1H, t, *J* = 2.1 Hz, H-6), 5.29 (1H, dd, *J* = 1.6 & 5.5 Hz, H-1″), 3.97 (1H, d, *J* = 10.8 Hz, H-2″) 3.84 (1H, s, H-5″), 3.46 (1H, s, H-3″), 3.13 (1H, d, *J* = 13.0 Hz, H-4″), 0.77–0.80 (1H, m, H-6″); ^13^C-NMR(175 MHz, DMSO-d_6_) δ 177.6 (C-4), 161.2 (C-7), 160.0 (C-4′), 157.1 (C-5), 156.5 (C-9), 147.2 (C-2), 134.1 (C-3), 130.6 (C-2′,6′), 120.5 (C-1′), 115.4 (C-3′,5′), 103.8 (C-10), 101.8 (C-1″), 98.9 (C-6), 93.8 (C-8), 71.1 (C-4″), 70.6 (C-3″), 70.3 (C-2″), 70.1 (C-5″), 17.5 (C-6″).

### Animals

Wild-type C57BL/6 male mice (4 weeks old; 18–20 g) were purchased from Orient Bio Inc. (Seungnam, Republic of Korea). The animals were housed in a room with a temperature of 23 ± 1 °C, a humidity of 50 ± 10%, a 12 h light/dark cycle, and free access to food and water. To decide the optimal dose of afzelin, we purchased the mice and acclimatized them for one week. They were randomly divided into six groups (n = 5–8); control, scopolamine + vehicle, scopolamine + 1 ng afzelin, scopolamine + 50 ng, scopolamine + 100 ng afzelin, and vehicle + 100 ng afzelin (afzelin only) groups. These mice were pre-treated with vehicle (PBS) or afzelin (1, 50, 100 ng/µl dissolved in PBS, three times a week) via cannulas into the third-ventricle of brains for one month. One month later, these pretreatment groups received 0.8 mg/kg scopolamine (dissolved in 0.9% saline, i.p.) and the control group and vehicle + 100 ng afzelin (afzelin only) groups were injected 0.9% saline (i.p.) before each behavior task. After we decided the optimal dose of the afzelin (100 ng), new mice were purchased and acclimatized to their new conditions for one week then they were randomly divided into three groups (n = 7–8); control, scopolamine + vehicle, and scopolamine + afzelin pretreatment group. All groups were administrated three times a week with either 0.5 μl of phosphate-buffered saline (PBS, as a vehicle) or 100 ng/µl afzelin dissolved in PBS for one month (In the first week, we did two times injection for acclimation then we injected three times a week for three weeks) (batch#2). One month later, scopolamine + vehicle and scopolamine + afzelin pretreatment groups received 0.8 mg/kg scopolamine (dissolved in 0.9% saline, i.p.) and the control group was injected 0.9% saline (i.p.) before each behavior task. During the behavioral tests, mice were treated (i.p.) with saline or scopolamine before each behavior task (30 min before). The schematic timeline displays in Fig. 3A. The Institutional Animal Care and Use Committee (IACUC) and the Institutional Biosafety Committee (IBC) at the Korea Institute of Science and Technology (KIST) approved all the procedures (Approval number, KIST-2019–048). All experiments were performed under relevant guidelines and regulations of the IACUC and the IBC in KIST.

### Cannulation of and chronic injection into the hypothalamic third ventricle

The hypothalamic third ventricle was cannulated as described^54,55^. We use ultraprecise small animal stereotactic apparatus (Kopf Instruments) to implant a 26-gauge guide cannula (Plastics One) at the midline coordinates of 2.0 mm posterior to the bregma and 5.0 mm below the skull. PBS, as a vehicle, and afzelin (three times a week) were injected via a cannula into the third ventricle of the brain for one month.

### Behavioral tests

All behavior tests were performed in the behavior testing room. An Anymaze video-tracking system (Stoelting) equipped with a digital camera connected to a computer was used. Following behavior tests were conducted. The study was carried out in compliance with the ARRIVE guidelines.

#### Passive avoidance test

The passive avoidance test was performed as described^56^. The apparatus (48 cm length × 23 cm width × 28 cm height) had a gate in the middle and was divided into two identical compartments, an illuminated (bright) compartment and a dark compartment. On the first day, the mouse was allowed to explore both compartments freely for 10 min. On the following day (training), the mouse was placed in the illuminated compartment for 60 s, then the gate was opened, allowing the mouse to freely enter the dark compartment. Once the mouse entered the dark compartment, the door was closed and the mouse’s foot was given an electric shock (0.3 mA, 3 s). The step-through latency, or time taken for the mouse to enter the dark compartment, was recorded. On the third day (probe trial), the mouse was placed in the illuminated compartment and then the gate was lifted after 60 s. The step-through latency was measured for 540 s and compared to the training day to gauge long-term memory.

#### Novel object recognition test

The object recognition test was conducted as described^7,57^. The apparatus was designed as a square arena (40 cm length × 40 cm width × 50 cm height). Before the first session, the mouse was allowed to explore the arena in the absence of any objects for 10 min (twice). During the first session (familiarization session), two identical objects were placed in the arena and the mouse was allowed to explore the objects for 20 min. During the second session (test session), one of the identical objects was replaced by a novel object the mouse was allowed to explore the objects for 10 min. The amount of time that the mouse spent exploring each object was recorded. A preference index was calculated using the ratio of the amount of time spent exploring any one of the two objects (including novel one) over the total time spent exploring both objects.

#### Y-maze test

The test was conducted as described previously^58^. A mouse was placed at the end of one arm of Y-maze which is made of three identical arms (40 cm length × 4 cm width × 15 cm height) with 120° angles between each arm. The mouse was allowed to move throughout the other arms for 10 min. Spontaneous alternations were measured from consecutive entries into all three arms (e.g., ABC, BCA, CAB, but not ABA), which were recorded. The alternation percentage was then calculated according to the following mathematical expression: [(number of alternations)/(total arm entries − 2)] × 100.

### Electrophysiology

The animals were chronically treated with afzelin into the third ventricle of the hypothalamus for one month at the dose of 100 ng/µl. Following the chronic administration of afzelin for one month, the animal was sacrificed and acute hippocampal slices were prepared (batch#1). The hippocampal slices were treated with standard aCSF containing either DMSO or scopolamine (100 μM) to the control carrying DMSO (Control), scopolamine + DMSO (Scop + vehicle), or scopolamine + afzelin (Scop + Afzelin) groups during the baseline recording (10 min) and for an additional 20 min after LTP induction by HFS. After that, the slices were treated with standard aCSF without drugs until the end of each experiment. The methods of hippocampal slice were described previously^59^. In brief, the hippocampi were quickly isolated from mice, and hippocampal slices (400 μm) were prepared using a vibratome (Leica, VT1000S). The cutting buffer contained 234 mM sucrose, 2.5 mM KCl, 1.25 mM NaH_2_PO_4_, 24 mM NaHCO_3_, 11 mM glucose, 0.5 mM CaCl_2_, 10 mM MgSO_4_, saturated with 5% CO_2_. For recovery, the slices were incubated at 35 °C for an hour in a recovery aCSF (artificial cerebrospinal fluid) solution containing 124 mM NaCl, 3 mM KCl, 1.25 mM NaH_2_PO_4_, 26 mM NaHCO_3_, 10 mM glucose, 6.5 mM MgSO_4_, 1 mM CaCl_2_, and maintained at room temperature thereafter. To evoke field excitatory postsynaptic potentials (fEPSPs) at the Schaffer collateral (SC)-CA1 synapses, the SC was stimulated with a 2-contact cluster electrode (FHC, CE2C55). The baseline fEPSPs were recorded at 0.033 Hz in a recording aCSF solution containing 124 mM NaCl, 3 mM KCl, 1.25 mM NaH_2_PO_4_, 26 mM NaHCO_3_, 10 mM glucose, 1.3 mM MgSO_4_, 2.5 mM CaCl_2_. Long-term potentiation (LTP) was induced using a high-frequency stimulation (HFS) protocol consisted of 100 Hz trains (1 s) repeated three times with a 30-s interval, and the fEPSP slope was monitored for 50 min following HFS. A MultiClamp 700B amplifier (Molecular Devices) with the pClamp10 software was used for data acquisition.

### Measurement of Ach level, AChE activity, and ChAT level in the hippocampus

The test was conducted as described previously^60^. The homogenized hippocampus was prepared for measurements of ACh/ChAT levels and AChE activities. The homogenized hippocampus was centrifuged at 12,000×g for 10 min at 4 °C. ACh levels activities were analyzed by the Amplex red ACh assay kit (Invitrogen Life Technologies, A12217, USA) with wavelengths of 560 nm and 590 nm, according to the manufacturer’s protocol. AChE levels activities were analyzed by the AChE Assay kit (BioChain, Z5030044, USA) with wavelengths of 412 nm, according to the manufacturer’s protocol. ChAT levels activities were analyzed by the ChAT Elisa kit (MyBioSource, MBS724080, USA) with wavelengths of 450 nm, according to the manufacturer’s protocol.

### Total RNA isolation, cDNA synthesis, and quantitative real-time PCR

The total RNA from the hippocampus tissue was extracted using TRIzol reagent (Invitrogen Life Technologies, USA) according to the manufacturer’s instructions. TRIzol reagent (1.0 mL) was added to each 50 mg of the hippocampus, then it was homogenized using a homogenizer. 0.2 mL of the chloroform was added to the lysate of the hippocampus with TRIzol reagent then it was incubated at room temperature. The supernatant was taken by centrifugation and 0.5 mL of isopropanol was added for the aqueous phase isolation. It was incubated at room temperature and centrifuged at 12,000 rpm (4 °C) for 10 min. A white gel-like pellet is a total RNA precipitated. 70% ethanol was added to this pellet and centrifuged at 12,000 rpm (4 °C) for 5 min for washing. After washing twice, the pellet, total RNA, was dissolved in diethylpyrocarbonate (DEPC)-treated water, and the concentration of total RNA was measured using the NanoDrop (Thermo Scientific). Complementary DNA was synthesized by reverse transcribing the total RNA (5 μg) using SuperScript III First-Strand Synthesis System for RT-PCR (Invitrogen). Complementary DNA (50 ng) was amplified using the Power SYBR Green PCR Master Mix kit (Applied Biosystems, USA) and primers with the following sequences: *Bdnf*, 5′-TCATACTTCGGTTGCATGAAGG-3′ and 5′-AGACCTCTCGAACCTGCCC-3′; *TrkB*, 5′- CTGGGGCTTATGCCTGCTG -3′ and 5′- AGGCTCAGTACACCAAATCCTA-3′; *Akt*, 5′- ATGAACGACGTAGCCATTGTG-3′ and 5′-TTGTAGCCAATAAAGGTGCCAT-3′; *Creb1*, 5′-AGCAGCTCATGCAACATCATC-3′ and 5′-AGTCCTTACAGGAAGACTGAACT-3′; and *Actb*, 5′-GGCTGTATTCCCCTCCATCG-3′ and 5′-CCAGTTGGTAACAATGCCATGT-3′. The StepOne Real-Time PCR System (Applied Biosystems, USA) for quantitative PCR (qPCR) was used for quantitative real-time PCR. PCR results were normalized to those of the control genes encoding β-actin (*Actb*).

### Western blot analysis

The hippocampi of the mice were homogenized on ice using RIPA buffer (Sigma). The homogenates were centrifuged at 16,000×g for 20 min, then the supernatant was used for this experiment. The protein concentration from this supernatant was determined by the Bradford protein assay (Bio-Rad). 30 µg of proteins were separated by 12% polyacrylamide gel electrophoresis and transferred to PVDF membranes (Millipore). After blocking in 5% skim milk, the membrane was incubated with rabbit anti-BDNF (1:800, Ab108319, Abcam) and mouse anti- β-actin (1:1000, Cell Signaling Technology) overnight at 4 °C. After the membranes were washed, they were incubated for one hour with HRP conjugated anti-rabbit (Abcam) or anti-mouse IgG antibody (Enzo Life Sciences). The blots were divided into two parts; β-actin staining and pro-BDNF/mature BDNF staining. The full-length images were provided in Supplementary Figs. S1 and S2. The bands were visualized using Image Quant LAS 4000 (GE Healthcare) with ECL reagent (Amersham), and the intensity was quantified using Image J software (National Institutes of Health).

### Immunofluorescence

The test was conducted as described previously^61,62^. Brains were removed and then placed in 4% paraformaldehyde overnight. The fixed brains were washed with PBS and dehydrated in 5–30% sucrose at 4 °C for 48 h. Each brain (20 μm thick) was sectioned with a cryostat (MICROM HM 525, Walldorf, Germany), then sections were blocked in PBT (0.05% Tween 20 in 1X PBS) containing 0.3% Triton X-100 (Sigma) and 3% donkey serum (Genetex) for one hour at room temperature. The following primary antibodies were used for immunofluorescence: mouse anti-CREB polyclonal antibody (1:500, 35-0900, Invitrogen), and. Brain sections were incubated with the primary antibodies at 4 °C overnight, and washed three times in PBT. Brain sections incubated for 2 h with Alexa Fluor 647-conjugated donkey anti-mouse secondary antibody (1:500, Invitrogen). After the incubated brain sections were washed three times with PBT, the sections were covered with Vectashield HardSet Antifade mounting medium with DAPI (Vector Laboratories, H‐1500) and imaged with a confocal microscope (LSM800; Carl Zeiss). For cell counting, the measurement of the analyzing particles using the Image J software was used to count specifically immuno-stained cells in the hippocampus.

### Statistical analysis

Experimental values were shown as mean ± standard error of the mean (S.E.M.) and evaluated with one-way ANOVA by Tukey’s post hoc test for Figs. 2, 3, 4, and 5 and one-way ANOVA by Dunnett’s post hoc test for Fig. 6 (quantifying immunofluorescence image). The statistical analysis was performed using the GraphPad PRISM software (GraphPad Prism Software Inc., version 8, CA, USA). P-values of < 0.05 were deemed significant.

## Supplementary Information

Supplementary Information.
