# Supplementary material for: Central administration of afzelin extracted from Ribes fasciculatum improves cognitive and memory function in a mouse model of dementia
Source: Sci Rep. 2021 Apr 28;11:9182. doi: 10.1038/s41598-021-88463-6 (PMC8080596; doi:10.1038/s41598-021-88463-6)
Supplement: Supplementary file 1 — Supplementary Information. [file 41598_2021_88463_MOESM1_ESM.pdf]

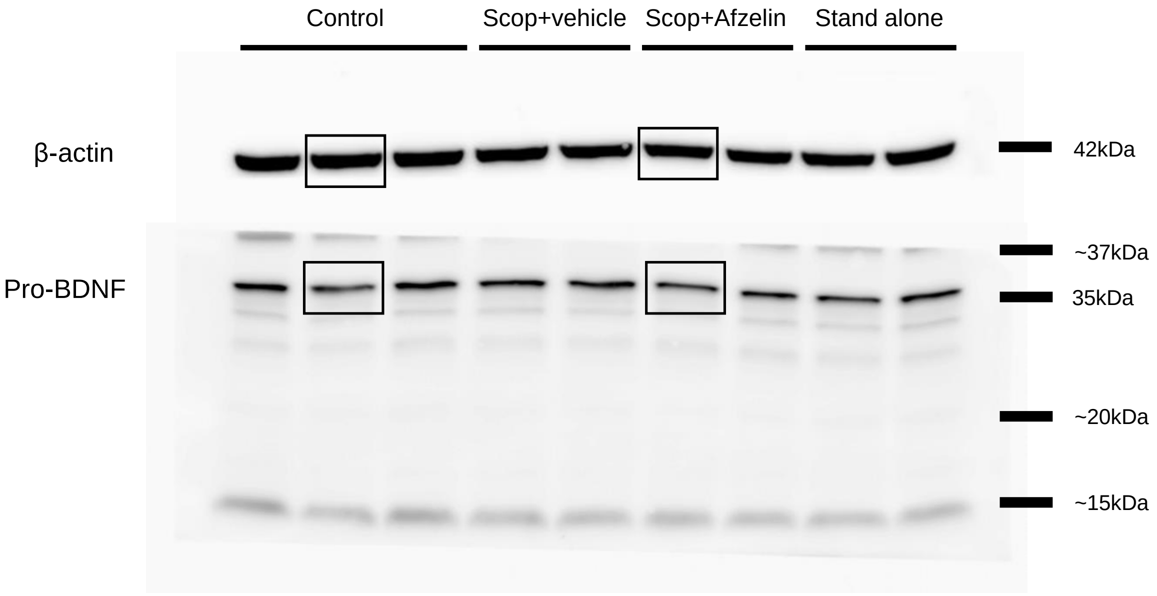

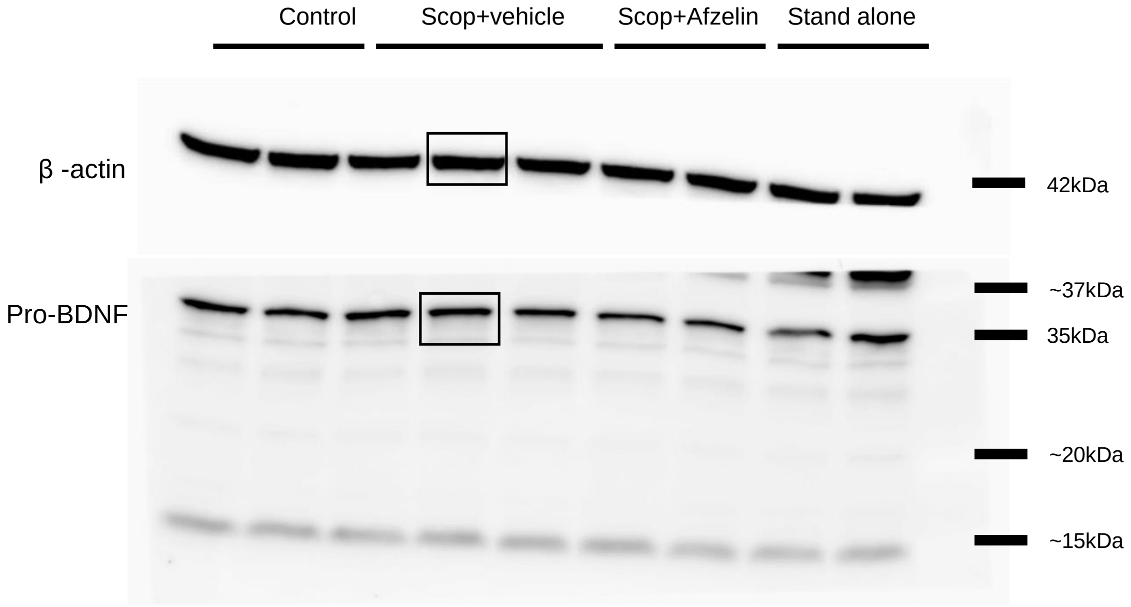

Supplementary figure 1. The images of full-length blots of pro-BDNA and β-actin proteins
Standard C57BL/6 mice (4 weeks old) were pre-treated into third-ventricle of brains cannula with 100 ng/µl of Afzelin (0.5 µl) or PBS (0.5 µl) for one month. The mice were received with scopolamine (Scop) injection (i.p. 0.8mg/kg) for the behavior tests then hippocampi of these mice were collected for measuring protein levels. The protein levels of β-actin and pro-BDNF were measured using western in the control (PBS as a vehicle), scopolamine+vehicle (Scop+vehicle, PBS as a vehicle), scopolamine+Afzelin (Scop+Afzelin, 100ng/µl afzelin of pretreatments) groups and vehicle+Afzelin (Veh+Afzelin, 100ng/µl afzelin of pretreatments) groups. The samples derived from the same experiment and that gels/blots were processed in parrellel. The black boxes were used for representative images for figure 5E.

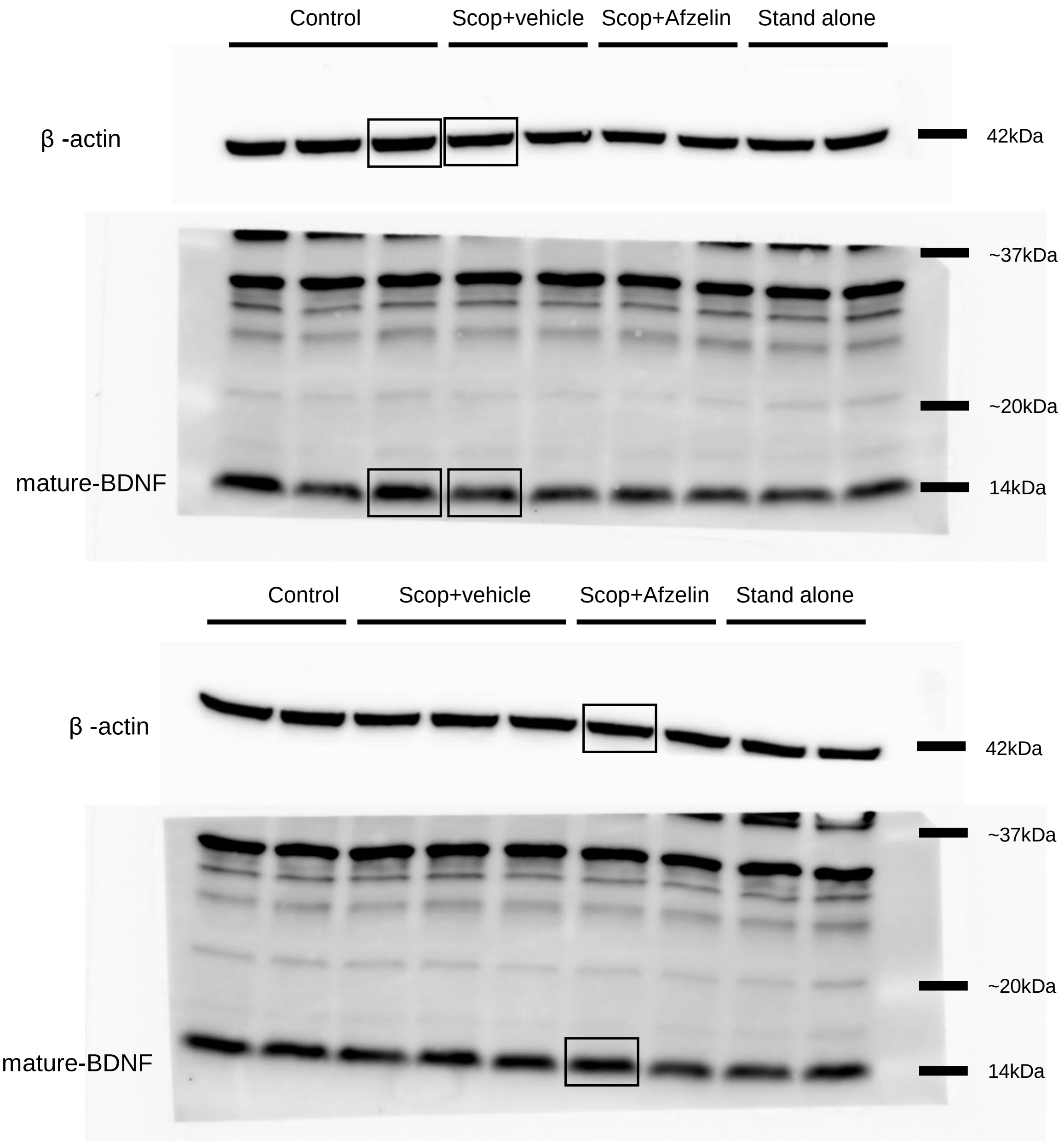

Supplementary figure 2. The images of full-length blots of mature BDNA and β-actin proteins
Standard C57BL/6 mice (4 weeks old) were pre-treated into third-ventricle of brains cannula with 100 ng/µl of Afzelin (0.5 µl) or PBS (0.5 µl) for one month. The mice were received with scopolamine (Scop) injection (i.p. 0.8mg/kg) for the behavior tests then hippocampi of these mice were collected for measuring protein levels. The protein levels of β-actin and mature BDNF were measured using western in the control (PBS as a vehicle), scopolamine+vehicle (Scop+vehicle, PBS as a vehicle), scopolamine+Afzelin (Scop+Afzelin, 100ng/µl afzelin of pretreatments) groups and vehicle+Afzelin (Veh+Afzelin, 100ng/µl afzelin of pretreatments) groups. The samples derived from the same experiment and that gels/blots were processed in parrellel. The black boxes were used for representative images for figure 5F.
